# Acute thrombotic occlusion involving three coronary arteries. A unique association with COVID‐19 pneumonia

**DOI:** 10.1002/ccr3.7803

**Published:** 2023-08-15

**Authors:** Mhd Baraa Habib, Mohamed Salah Abdelghani, Ahmed Elyas, Anas A. Ashour, Mohammad Altermanini, Shahid Imran, Abdul Rahman Arabi

**Affiliations:** ^1^ Department of Internal Medicine Hamad Medical Corporation Doha Qatar; ^2^ Cardiology Department, Heart Hospital Hamad Medical Corporation Doha Qatar

**Keywords:** coronary artery disease, COVID‐19, myocardial infarction, SARS‐CoV‐2, thrombosis

## Abstract

**Key Clinical Message:**

Respiratory viruses, particularly COVID‐19, can be associated with severe cases of myocardial infarction (MI). Physicians should have a low threshold for MI in COVID‐19 patients who present with persistent chest pain as MI in rapidly deteriorating cases can be missed. Prompt response includes both timely diagnosis and swift treatment.

**Abstract:**

The coronavirus disease 2019 (COVID‐19) is associated with coronary artery thrombosis. Many cases of single‐vessel and few cases of two‐vessel thrombosis were reported. Herein, we report a unique association in a middle‐aged man diagnosed with COVID‐19 and presented later with acute myocardial infarction causing cardiogenic shock. The patient was found to have three‐vessel thrombosis.

## Graphical abstract

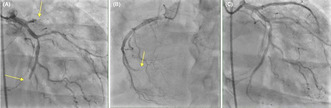

## 1 INTRODUCTION

Coronaviruses are large single‐stranded RNA viruses known to cause many human and animal diseases.^1^ In December 2019, multiple cases of pneumonia were recognized in Wuhan, China.^2^ The pathogen responsible for causing these clinical conditions was later identified as a novel member of the coronaviruses family, that was named severe acute respiratory syndrome coronavirus 2 (SARS‐CoV‐2).^2^ As the disease has since spread rapidly in China and globally, the World Health Organization (WHO) designated the disease as a pandemic in March 2020.^3^ By May 2022, coronavirus disease 2019 (COVID‐19) has infected more than 500 million persons and has led to more than 6.2 million deaths.^4^ The disease can be asymptomatic or cause a variety of symptoms that ranges from mild to critical. The most frequent symptoms are fever, cough, myalgia, headache, and dyspnea.^5^ However, cardiovascular manifestations including arrhythmia, pulmonary embolism, ischemic strokes, myocarditis, and myocardial infarction (MI) were also reported.^6^

It has been well established that respiratory viral infections can trigger MI.^7^ In a review of 26 studies including more than 11,000 COVID‐19 patients, the pooled prevalence of acute myocardial injury evident by elevation of cardiac biomarkers (high‐sensitivity troponins and creatine kinase MB) was found to be around 20%, and appeared to be associated with disease severity and prognosis.^8^ Several studies and systematic reviews described the relationship between COVID‐19 and the increased risk of thrombosis.^9^, ^10^, ^11^ It was found that thrombosis plays a major role in the morbidity and mortality of the disease.^9^, ^10^, ^11^ In addition, multiple reports described an association between COVID‐19 and coronary artery thrombosis.^12^, ^13^, ^14^, ^15^ However, only few cases described simultaneous two‐coronary vessels acute thrombosis.^16^, ^17^ Herein, we present a case of a middle‐aged man who was diagnosed with COVID‐19 infection and presented again with severe acute chest pain. The patient was found to have MI due to an acute three‐vessel thrombosis.

## 2 CASE REPORT

**FIGURE 1:**
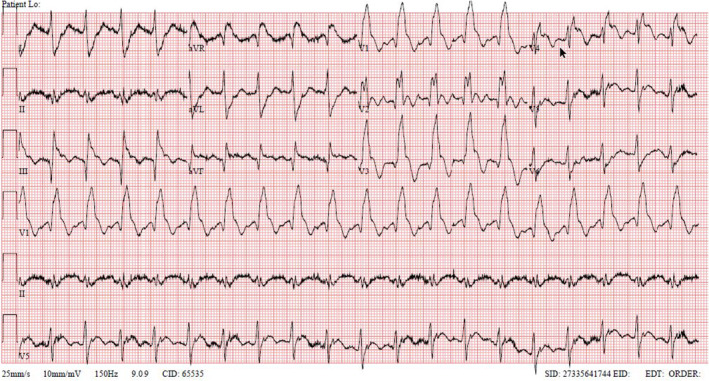
ECG upon admission: Sinus tachycardia with right bundle branch block and ST‐segment elevation in V1‐4 (shark fin pattern), lead III, and aVF, with prominent ST‐segment elevation in aVR as well.

A 47‐year‐old male known to have hypertension and Type‐II diabetes mellitus, presented with dry cough, fever, and shortness of breath for 10 days. The patient denied having chest pain, palpitations, or any other symptoms. The patient was nonsmoker and with no family history of coronary artery diseases. Physical exam was only significant for bilateral fine crackles. COVID‐19 nasopharyngeal swab was done and came back positive. The patient was discharged for home isolation with symptomatic treatments. Six days later, the patient presented again with progressive shortness of breath associated with central compressive chest pain which was continuous and increasing in severity for the last few hours. On presentation, the patient was in pain and distress with initial oxygen saturation of 77% on room air. Blood pressure was 174/93 mmHg, and heart rate was 119 beats/minute. Chest examination revealed diffuse bilateral fine crackles only. A 12‐lead electrocardiogram (ECG) showed ST‐segment elevation in aVR, anterior and inferior leads (Figure 1). The patient was diagnosed with acute ST‐segment elevation myocardial infarction (STEMI). He was loaded with aspirin 300 mg and clopidogrel 600 mg then placed on continuous positive airway pressure therapy (CPAP). Laboratory tests that were sent on presentation showed high levels of troponin and D‐dimer (Table 1).

**TABLE 1:** Blood tests upon admission.

Detail	Value w/units	Normal range
Platelet	306 × 10^3/uL	150–400
WBC	14.1 × 10^3/uL	4.0–10.0
Hgb	14.8 gm/dL	13.0–17.0
Creatinine	120 umol/L	62–106
NT pro‐BNP	8612 pg/mL	
CRP	382.9 mg/L	0.0–5.0
D‐Dimer	98.10 mg/L FEU	0.00–0.49
pH Ven‐POC	7.401	7.320–7.420
Troponin‐T HS (upon admission)	327 ng/L	3–15
Troponin‐T HS (after 10 h)	39,325 ng/L	3–15
Troponin‐T HS (after 16 h)	71,873 ng/L	3–15
HbA1C %	7.7%	
Cholesterol	3.4 mmol/L	
Triglyceride	2.4 mmol/L	
HDL	0.4 mmol/L	
Ferritin	13,072.0 ug/L	30.0–490.0
Interleukin‐6	108 pg/mL	

**FIGURE 2:**
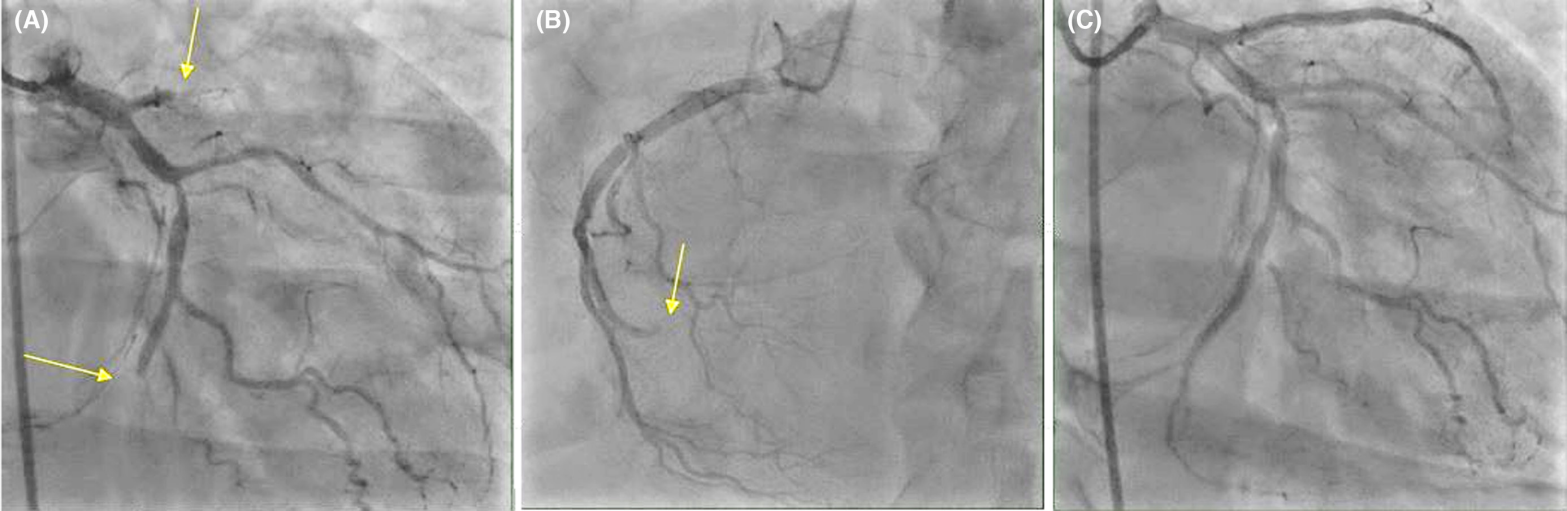
**(**A) Proximal left anterior descending artery (LAD) 100% thrombotic occlusion and distal Left circumflex artery (LCX) 100% thrombotic occlusion. (B) distal right coronary artery (RCA) 100% thrombotic occlusion. (C) Post‐percutaneous coronary intervention to LAD and LCX.

**FIGURE 3:**
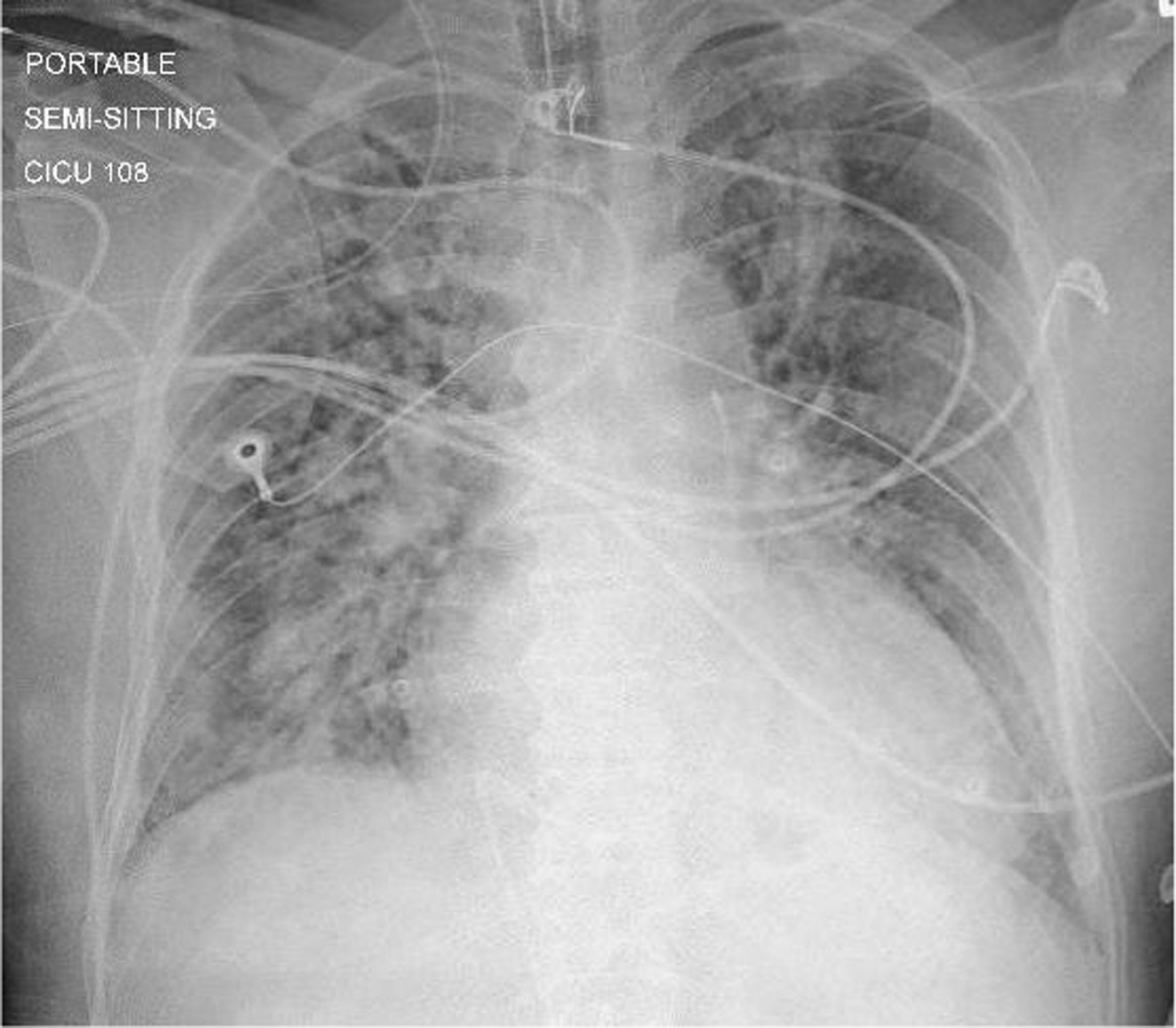
Chest x‐ray: Bilateral accentuation of lung reticulation and interstitial markings with non‐homogenous infiltrates mainly in the right lung parenchyma and to the lesser extent in the left infraclavicular region.

Soon after presentation, the patient was intubated. However, immediately after intubation, he developed cardiac arrest with pulseless electrical activity. Cardiopulmonary resuscitation (CPR) was started and return of spontaneous circulation was achieved after 1 cycle of CPR. Again, the patient developed ventricular fibrillation which reverted with defibrillation. Therefore, he was started on amiodarone infusion and transferred for a primary percutaneous coronary intervention (PCI). Coronary angiography (CAG) revealed a three‐vessel disease with acute thrombotic occlusion in left anterior descending artery (LAD), left circumflex artery (LCX) and distal right coronary artery (RCA). PCI was done to proximal‐mid LAD and distal LCX with good final result of TIMI‐3 flow (Figure 2). As the patient was in an unstable condition and the RCA lesion was distal, it was planned for a relook PCI once the patient condition stabilizes. Hemodynamic studies showed a preserved cardiac output and cardiac index, a moderately elevated pulmonary capillary wedge pressure, and a severely elevated right ventricular filling pressure. Chest x‐ray showed bilateral patchy infiltrates consistent with COVID‐19 pneumonia (Figure 3). The patient was sent to cardiac intensive care unit for close observation and management. The prescribed medications included eptifibatide for 18 hours after PCI, aspirin, clopidogrel, heparin, and furosemide. He also received COVID‐19 pneumonia treatment protocol including dexamethasone. However, over the next few days, the patient developed septic shock and multiorgan failure. The patient passed away despite maximal supportive therapy and broad‐spectrum antibiotics.

## 3 DISCUSSION

While it became well‐known that COVID‐19 is linked to increased thrombosis including MI, patients usually present with a single‐vessel, or in rarer occasions, two‐vessel MI.^12^, ^13^, ^16^, ^17^ In our case, the patient presented with acute MI and found to have an acute thrombotic occlusion involving all three main coronary arteries simultaneously. This represents a unique association with COVID‐19 pneumonia. His presentation was preceded with COVID‐19 coryzal symptoms for few days denoting that the infection was probably the culprit for his acute thrombosis. Acute arterial thromboembolic disease was assessed in a retrospective multicentric study which revealed acute coronary occlusions in 9 out of 24 patients.^18^ Thrombotic events were mainly observed in non‐atherosclerotic arteries and occurred in younger patients.^18^ COVID‐19 was also associated with dense thrombus burden, higher rates of multivessel thrombosis, stent thrombosis, and poorer outcome as concluded from an observational British study involving 115 consecutive STEMI patients.^15^ Furthermore, multivessel thrombosis was observed in 18% of COVID‐19 STEMI patients compared to none in COVID‐19–negative group.^15^ In another multicenter retrospective study which included 78 patients, there was an alarming rate of stent thrombosis which has occurred in 4 (21%) out of 19 patients who had PCI.^19^ This study was conducted in the beginning of the pandemic from February till April 2020 and probably before the widespread use of anticoagulation in patients with high D‐dimer. Moreover, one case report showed significant thrombus burden after use of thrombolytic therapy in a STEMI patient, eventually requiring thrombectomy and stent placement.^20^ In a review of coronary thrombosis management strategies, multiple challenges were raised including the delay in door‐to‐balloon time, drug–drug interaction, drug–disease interaction, and efficacy of anticoagulants therapy.^21^ In addition, the virus is linked to high thrombogenicity evident by multiple case reports of high thrombus burden and high risk of stent thrombosis.^21^ Moreover, some cases also reported cardiac chamber thrombi.^22^, ^23^

Although studies showed a significant association between COVID‐19 and risk of thrombosis, the exact mechanism is not fully understood. Several mechanisms of thrombosis were suggested in COVID‐19 patients including immune thrombosis triggered by cytokine storms that can indirectly initiate coagulation activation and thrombin generation.^11^ This process can eventually lead to disseminated intravascular coagulation (DIC).^11^ In contrary to DIC caused by other diseases, COVID‐19 infection is not commonly associated with major bleeding and is usually associated with high fibrinogen and factor VIII activity.^24^ This highlights that COVID‐19‐associated DIC mechanism is more comparable to chronic DIC as compared to acute DIC.^24^ Another possible mechanism of the relationship of COVID‐19 and thrombosis is the direct invasion of the virus to the endothelial cells causing cell injury and thrombosis.^25^

## 4 CONCLUSION

COVID‐19‐related thrombophilia became well‐known, and recently described as a potential reason behind the thromboembolic events including coronary artery thrombosis. However, simultaneous three‐vessel coronary artery thrombosis is a unique finding that raises the alarm. Physicians should have a low threshold for MI in COVID‐19 patients who present with persistent chest pain as MI in rapidly deteriorating COVID‐19 patients can be missed. Diagnosis with ECG and cardiac enzymes as well as treatment with PCI and medications could improve patient prognosis if done promptly. In the post‐COVID‐19 pandemic era, similar attention should focus on the association of other respiratory viral infections with MI. Further research is needed to explore the exact process of high thrombogenic tendency of COVID‐19 infection and dedicated studies are recommended to look for management strategies in those patients.

## AUTHOR CONTRIBUTIONS

**Mhd Baraa Habib:** Conceptualization; investigation; methodology; resources; writing – original draft; writing – review and editing. **Mohamed Salah Abdelghani:** Conceptualization; resources; visualization; writing – review and editing. **Ahmed Elyas:** Conceptualization; supervision; validation; writing – review and editing. **Anas A. Ashour:** Conceptualization; funding acquisition; writing – original draft; writing – review and editing. **Mohammad Altermanini:** Conceptualization; writing – original draft; writing – review and editing. **Shahid Imran:** Conceptualization; supervision; validation; writing – review and editing. **Abdul Rahman Arabi:** Conceptualization; project administration; supervision; writing – review and editing.

## FUNDING INFORMATION

This research did not receive any specific grant from funding agencies in the public, commercial, or not‐for‐profit sectors.

## CONFLICT OF INTEREST STATEMENT

The authors report no conflict of interest.

## ETHICS STATEMENT

The case was approved for publication by Hamad Medical Corporation IRB with a protocol number MRC‐04‐21‐374.

## CONSENT FOR PUBLICATION

Written informed consent was obtained from the patient to publish this report in accordance with the journal's patient consent policy.

## Data Availability

Data sharing is not applicable to this article as no datasets were generated or analyzed during the current study.
